# Anterior Mediastinal Mature Teratoma Focally Infiltrating Pulmonary Parenchyma

**Published:** 2013-11-18

**Authors:** Angelina Vaccaro, Francesco Vierucci, Francesca Dini, Silvia Ruggieri, Laura Crespin, Lucia Matteucci, Raffaele Domenici

**Affiliations:** Pediatric Unit, Campo di Marte Hospital, Lucca, Italy

**Dear Sir,**

Teratomas are germ cell tumors. Mediastinum is the second most common extra-gonadal site of these tumors.[1-3] Mediastinal masses in children and adolescents are usually considered malignant. Even benign teratomas have been reported to transform to malignant ones in case of delayed diagnosis or presentation.[3-5] Moreover, large mediastinal teratoma without any long-term symptomatology is quite rare. We describe here a large anterior mediastinal mature teratoma diagnosed in a paucisymptomatic adolescent.

A 15-years-old girl was admitted for a two-days-long history of low-grade fever associated with acute-onset of cough and mild thoracic pain. Pain was referred to the left hemithorax and increased with breathing and movements. Vital signs were normal, with a room-air oxygen saturation level of 99%. Physical examination was unremarkable, particularly with normal breath sounds at chest auscultation. Unexpectedly chest x-ray revealed a large homogeneous mediastinal paracardiac mass in the left hemithorax (Fig. 1). Echocardiogram showed normal cardiac function with mild pericardial effusion. Contrast enhanced computed tomography (CT) scans confirmed a well-demarcated mediastinal partially cystic mass of about 11x9x8 cm with regular borders, completely enclosing the thymus and containing soft tissue, fat and a small calcification (Fig. 2,3). The mass mildly compressed the adjacent structures. There were no radiological signs of invasion into the surrounding mediastinal structures. These radiological findings were consistent with the diagnosis of cystic teratoma. Biochemical evaluation showed lymphocytosis with neutrophilia (white blood cell count 15,240/mm3, neutrophils 67%) and increased ESR (67 mm/1sth). Alpha-fetoprotein and beta human chorionic gonadotropin were within normal limits. At left postero-lateral thoracotomy, the mass occupied the anterior mediastinum and focally infiltrated pulmonary parenchyma of the inferior lobe of the left lung. The mass and the infiltrated part of the inferior lobe of the left lung were totally excised. Histopathological analysis confirmed the diagnosis of mature cystic teratoma without immature elements. Chemotherapy was not administered. After a one-year of clinical, biochemical and radiological follow-up no recurrence of disease was evident. Long term follow-up is in plan.

**Figure:**
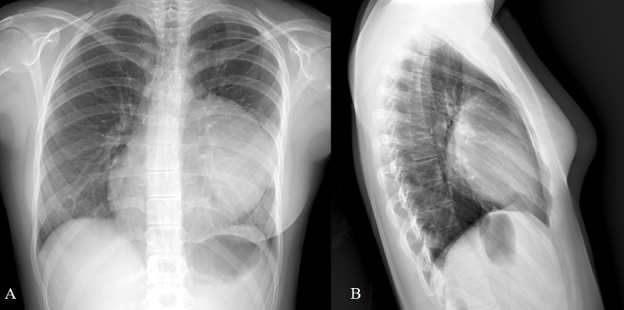
Figure 1: PA (A) and Lateral (B) chest X-rays showing a large homogeneous soft tissue mass.

**Figure:**
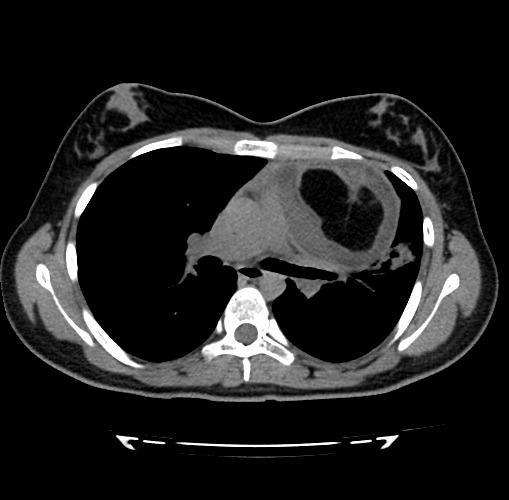
Figure 2: Axial CT scan showing a well-demarcated multi-septated cystic mass compatible with mediastinal teratoma.

**Figure:**
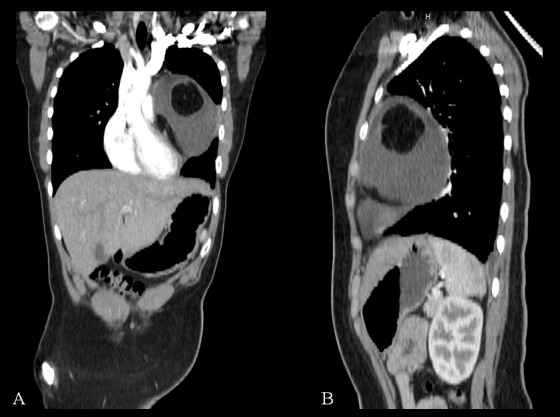
Figure 3: Coronal (A) and sagittal (B) CT reconstructions of mediastinal cystic teratoma. The mass appeared to compress the adjacent structures without invasion. A calcification is evident in coronal reconstruction.

Up to 80% of pediatric mediastinal teratomas are benign, with 20% revealing immature elements by histological evaluation.[1,2] Naturally occurring malignant transformation of mediastinal teratoma is rare, but long-standing, mature teratomas may be at risk of malignant change during childhood and adolescence [4, 5]. In a recent review Paliwal et al suggested that mature mediastinal teratomas had increased risk of malignant transformation in adenocarcinoma when the tumor was at least 10 cm in size or in presence of areas of thickening.[4] Takahashi et al. reported a case of an anterior mediastinal teratoma with malignant transformation in embryonal rhabdomyosarcoma in a 12-year-old-boy.[5] Dimensions and margins of mediastinal teratomas and the possible presence of areas of thickening should always be assessed to suspect malignant transformation.

Mediastinal teratomas produce non-specific symptoms (mainly chronic cough, dyspnoea and wheezing or severe respiratory distress due to airway compression) when they attain large size or may rupture into the lung and bronchial tree, pleural space, pericardial space, or erode great vessels.[2,3,6]. In our case, the patient reported with acute-onset of febrile illness with unremarkable past history and physical examination. The referred mild thoracic pain induced us to perform chest x-ray allowing the detection of a large mediastinal mass. Chest x-ray is usually able to identify mediastinal masses up to 90% of cases.[7] However, CT is the modality of choice to establish the nature of the mass (solid or cystic), the location and its relationship to the adjacent structures.[2]

Complete surgical resection is the treatment of choice for mediastinal teratomas followed by an extensive histological examination.[8] Postoperative chemotherapy is required in malignant tumors.[1] However, long-term clinical, biochemical and radiological surveillance is necessary.[8] In conclusion, mediastinal teratomas are rare and usually benign, but long term follow-up is required to detect recurrences.

## Footnotes

**Source of Support:** Nil

**Conflict of Interest:** None declared

